# Inflammageing and Cardiovascular System: Focus on Cardiokines and Cardiac-Specific Biomarkers

**DOI:** 10.3390/ijms24010844

**Published:** 2023-01-03

**Authors:** Marco Alfonso Perrone, Alberto Aimo, Sergio Bernardini, Aldo Clerico

**Affiliations:** 1 Department of Cardiology and CardioLab, University of Rome Tor Vergata, 00133 Rome, Italy; 2 Fondazione CNR-Regione Toscana G. Monasterio and Scuola Superiore Sant’Anna, 56127 Pisa, Italy; 3 Department of Experimental Medicine, University of Rome Tor Vergata, 00133 Rome, Italy

**Keywords:** ageing, inflammation, cardiovascular risk, cardiac natriuretic peptides, cardiac troponins, myocardial injury, acute myocardial infarction

## Abstract

The term “inflammageing” was introduced in 2000, with the aim of describing the chronic inflammatory state typical of elderly individuals, which is characterized by a combination of elevated levels of inflammatory biomarkers, a high burden of comorbidities, an elevated risk of disability, frailty, and premature death. Inflammageing is a hallmark of various cardiovascular diseases, including atherosclerosis, hypertension, and rapid progression to heart failure. The great experimental and clinical evidence accumulated in recent years has clearly demonstrated that early detection and counteraction of inflammageing is a promising strategy not only to prevent cardiovascular disease, but also to slow down the progressive decline of health that occurs with ageing. It is conceivable that beneficial effects of counteracting inflammageing should be most effective if implemented in the early stages, when the compensatory capacity of the organism is not completely exhausted. Early interventions and treatments require early diagnosis using reliable and cost-effective biomarkers. Indeed, recent clinical studies have demonstrated that cardiac-specific biomarkers (i.e., cardiac natriuretic peptides and cardiac troponins) are able to identify, even in the general population, the individuals at highest risk of progression to heart failure. However, further clinical studies are needed to better understand the usefulness and cost/benefit ratio of cardiac-specific biomarkers as potential targets in preventive and therapeutic strategies for early detection and counteraction of inflammageing mechanisms and in this way slowing the progressive decline of health that occurs with ageing.

## 1. Introduction

The average lifespan of humans is increasing, and with it the percentage of people entering the 65 and older age group is growing rapidly and will continue to do so in the next 20 years [1,2,3,4,5,6,7,8]. Between 2019 and 2050, the number of persons aged sixty-five or over globally is projected to more than double, while the number of children under five is to remain relatively unchanged. Consequently, this projection indicates that in 2050, there will be more than twice as many older persons as children under five [9].

It has been evaluated that life expectancy at birth would increase by about six years if all heart and cardiovascular diseases were cured [9]. Cardiovascular diseases typically manifest clinically after the fifth or sixth decade of life; however, there is a high interindividual variability in disease onset and associated mortality [5,8,9]. Several clinical studies, based on selected populations with exceptional longevity, have supported the evidence that individuals do not age at the same pace [6,7,10,11]. Accordingly, some authors suggested the use of the word “biological aging” (or functional or physiological aging) rather than the chronological age [6,7,10,11].

Experimental and clinical studies are essential to develop early-life biomarkers that efficiently identify individuals who are at high risk of developing accelerated heart and vascular damage, with the ultimate goal of improving primary prevention and reducing the health care and socioeconomic impact of age-related cardiovascular disease. The search for reliable indicators of biological age has been ongoing for over three decades, largely without success until recently [10,11]. Oxidative stress biomarkers and proinflammatory cytokines have been usually reported to be involved in age-related conditions, including cardiovascular diseases [10,11,12,13]. The term “inflammageing” was introduced by Franceschi et al. in 2000 (1). Inflammageing is typical of elderly individuals and is characterized by a combination of elevated levels of inflammatory biomarkers, a high burden of comorbidities, an elevated risk of disability, frailty, and premature death [1,2,3,4]. Several recent studies have demonstrated that inflammageing is a hallmark of various cardiovascular diseases, including atherosclerosis, hypertension, and rapid progression to heart failure [3,4,5,6]. Furthermore, recent clinical studies have demonstrated that cardiac-specific biomarkers, cardiac natriuretic peptides and cardiac troponins (cTn), are able to identify patients at highest risk of progression to heart failure [14,15].

The principal aim of this review is to discuss the role of circulating biomarkers in detecting and monitoring the adverse effects of ageing mechanisms on cardiovascular system. First, the oxidative stress will be introduced as the principal pathophysiological mechanism related to inflammageing, then the analytical and pathophysiological aspects related to the evaluation of the total oxidative status and the specific markers of oxidative stress will be briefly discussed. Later, the pathophysiological role of circulating markers (and especially of cardiac-specific biomarkers) will be discussed in detail. Finally, the clinical relevance of hs-cTnI and hs-cTnT assay for an early and accurate detection of asymptomatic individuals at higher risk of progressing to symptomatic heart failure (HF) or developing major adverse cardiovascular events (MACE) will be suggested [15,16].

## 2. Evaluation of Oxidative Stress

In Table 1, the authors have reported the most important mechanisms of inflammageing process related to cardiovascular system in accordance with the literature data [3,4,6,7,13,14]. Several studies support the important role of oxidative stress in reducing life span duration from invertebrate to vertebrate animals [6,7,13,14,17,18,19,20,21,22,23,24,25,26,27,28,29,30,31,32,33,34]. However, Pérez et al. [35] reported that the overexpression of some antioxidant enzymes, such as copper zinc superoxide dismutase (CuZnSOD), catalase, or combinations of either CuZnSOD and catalase or CuZnSOD and manganese superoxide dismutase (MnSOD), which are known to scavenge superoxide and hydrogen peroxide in the cytosolic and mitochondrial compartments, are insufficient to extend lifespan in mice.

As for the studies in humans, lower levels of markers of oxidative stress and/or increased levels of antioxidant molecules have been reported in very elderly individuals compared with younger individuals [6]. In particular, Paolisso et al. [36] measured, in plasma samples collected from Italian centenarians, some indices of oxidative stress (specifically reaction products of malondialdehyde with thiobarbituric acid) and lipid hydroperoxides, and also plasma concentrations of antioxidant defenses (i.e., plasma vitamin E, vitamin C, and reduced/oxidized glutathione ratio). The degree of oxidative stress was lower in 22 healthy centenarians than in subjects aged ≤99 years (including 30 adults <50 years and 30 with 70–99 years) [36]. Mecocci et al. [37] reported higher levels of antioxidant vitamins C and E and lower levels of reaction products of malondialdehyde with thiobarbituric acid and lipid hydroperoxides in the plasma of 32 Italian healthy centenarians compared with elderly participants of younger age.

Low doses of reactive oxygen species (ROS) may exert such beneficial effects, whereas higher doses are unquestionably detrimental [6,18]. This biphasic responses of biological organisms to a potentially harmful compound are commonly named “hormesis”, as initially postulated in 1943 by Southam and Ehrlich [38]. The mitohormesis phenomenon may have significant impact on aging with a variety of stressors [6,18,38,39,40,41,42].

More specifically, some circulating biomarkers related to oxidative stress have been suggested as reliable indexes of inflammageing mechanisms related to human cardiovascular diseases [43,44,45,46,47,48,49]. According to Marocco et al. [45] several methodological approaches have been used for the assessment of oxidative status in humans, including: (1) ROS in leukocytes and platelets by flow cytometry; (2) markers based on ROS-induced modifications of lipids, DNA, and proteins; (3) enzymatic players of redox status; (4) total antioxidant capacity of human body fluids. In particular, a list of the most commonly used biomarkers may include: malondialdehyde (MDA), uric acid, HDL-C, superoxide dismutase (SOD), glutathione peroxidase (GPX), aldehyde DNA damage, protein radicals, hydrogen peroxide, markers of lipid peroxidation, transcription factor NF-κB, cyclooxygenase-2, and catalase [43,44,45,46,47,48,49].

Even if several markers of oxidative stress are usually used at the same time, the results obtained with different methods for the same or among different markers do not correlate well with each other or do not fully reflect the state of oxidative stress [45,46,50,51]. Furthermore, there is a lack of consensus concerning the validation, standardization, and reproducibility of methods currently used for the measurement of markers of oxidative stress [45].

### 2.1. Evaluation of Total Oxidative Status

To overcome the problems related to measurement of only one marker of oxidative stress, a commonly used analytical approach consists of the evaluation of the Total Antioxidant Capacity (TAC) of a solution, as evaluated either by determining the rate of oxidation of the antioxidant or by measuring the protection of an easily determined indicator against oxidation by the antioxidants [45,51,52,53]. The TAC, also named “the nonenzymatic antioxidant capacity” (NEAC), is defined as the moles of oxidants neutralized by one liter of body fluids and is commonly used to test the oxidative potential of some vegetable foods, but also to investigate oxidative stress in many pathological conditions in humans [45,51,52,53,54]. In plasma, nonenzymatic antioxidants include endogenous (e.g., uric acid, bilirubin, and thiols) and nutritional (e.g., tocopherols, ascorbic acid, carotenoids, and phenolics) compounds [51,52,53]. From an analytical point of view, some assays for TAC measure either their radical scavenging or reducing capacity [45,50,51,55,56,57,58,59,60,61,62,63,64,65].

An alternative procedure is to calculate an index between the ratio of oxidized and reduced status of several substances in biological fluids, especially serum (or plasma) and urine samples [45,66,67]. In particular, the OXY-SCORE [66] was evaluated by subtracting the protection score (including the valuation of reduced glutathione, alpha- and gamma-tocopherol levels, and antioxidant capacity) from the damage score (including the evaluation of plasma free and total malondialdehyde, reduced/oxidized glutathione ratio, and urine F2-isoprostanes). To calculate the OXY-SCORE, plasma free and total malondialdehyde (F- and T-MDA), glutathione disulphide/reduced (GSSG/GSH) and urine isoprostanes (iPF2alpha-III) levels were combined as oxidative damage markers (damage score) [66].

An alternative method is to evaluate the Oxidative-INDEX [67,68,69]. The Oxidative-INDEX can be calculated by subtracting the results of OXY test from those of ROMs test. In brief, the total antioxidant capacity can be estimated by evaluating capacity of each sample to inactivate the oxidant solution (HClO) added in excess [67,68,69]. The d-ROMs test is based on the reaction of serum samples with transition metal ions to form alkoxy and peroxy radicals, each sample being added to a reaction mixture, obtained after addition of N,N-diethyl-para-phenylendiamine to pH 4.8 acetate buffer [67,68,69]. The Oxidative-INDEX based on the evaluation of serum hydroperoxides (ROMs) and total antioxidant capacity (OXY) using a colorimetric assay (named d-ROMs and Oxy-adsorbent Tests, Diacron, Italy) is commercially available for the evaluation of redox status in experimental animals and humans [66,67,68,69,70,71].

The indexes of oxidative stress status can be evaluated using simple and cheap colorimetric assay methods. Several studies have suggested that OXY-SCORE and Oxidative-INDEX may be clinically useful because the results are correlated to age, gender, physical exercise, smoking habit, and some clinical conditions, including cardiovascular disease [66,67,68,69,70,71,72]. However, these indexes do not provide information about the mechanisms altering the oxidative status. This information can be obtained from biological substances that can be modified by pathophysiological mechanisms specifically related to oxidative stress.

### 2.2. F2-Isoprostanes (F_2_-IsoPs)

Biomarkers of oxidative stress are usually classified as molecules modified by interactions with ROS and as molecules of the antioxidant system that become changed in response to increased redox stress [43,44,45,46]. A plethora of biomarkers has been used, but many of these do not correlate well with each other or do not fully reflect the oxidative state [43,44,45,46]. Furthermore, most markers lack sensitivity or specificity or require invasive techniques. For these reasons, the search is in progress for new substances which can better fit the characteristics of an ideal biomarker, as summarized in Table 2 [12,73].

The measurement of F2-isoprostanes (F_2_-IsoPs) should be considered the most reliable approach to assess oxidative stress in vivo [43,44,74,75,76,77,78]. F_2_-IsoPs are a family of stable, prostaglandin-like compounds generated from the peroxidation of arachidonic acid, a polyunsaturated fatty acid present in phospholipids of cell membranes [74,75]. The generation of F_2_-IsoPs from arachidonic acid is independent of the cyclooxygenase enzyme that catalyzes the formation of prostaglandins from arachidonic acid [74,75]. F_2_-IsoPs have been shown to exert biological effects via receptor-dependent and independent mechanisms and might serve as mediators of oxidant injury [75].

Over the last 30 years, a multitude of papers has been published describing different analytical methods for the quantification of F_2_-IsoPs in biological fluids [79]. The methods used are: (1) gas chromatography–mass spectrometry (MS), (2) liquid chromatography–MS, and (3) immunoassays (ELISA). MS-based assays are considered more accurate than immunoassay methods. Indeed, the antibodies currently used in immunoassay methods are not completely specific for F_2_-IsoPs, due to some structural similarities between F_2_-IsoPs and some COX-derived prostaglandins as well as other related molecules [79,80,81,82,83]. F_2_-IsoPs can be measured in plasma, urine, any tissue, cerebral spinal fluid, exhaled breath condensate, amniotic fluid, and saliva [75,77,79]. Measurement in plasma and urine is most common in humans as these fluids are the ones most easily sampled. Caution must be taken when collecting and storing plasma F_2_-IsoPs as these molecules can be generated from ex vivo oxidation of arachidonic acid in the plasma. Some authors suggested that all samples should be stored at −80 °C, not −20 °C, upon collection because autoxidation can even occur at −20 °C, leading to artifactual generation of F_2_-IsoPs during storage [84].

F_2_-IsoPs have been shown to be a reliable biomarker of endogenous lipid peroxidation because they are ubiquitous in the body and are chemically stable in biological fluids when stored correctly [44,79]. Elevated F_2_-IsoPs are also found in a wide range of disorders [79]. In particular, elevated concentrations of F_2_-IsoPs are found in patients with cardiovascular diseases, correlate with the extent of the disease, and can predict outcome [78]. In 2013, a meta-analysis reported that 20 studies out of 22 showed a significant association between F_2_-IsoPs and cardiovascular disease, but data on prediction of future cardiovascular events are lacking, because only two articles provided data from prospective studies [85]. F_2_-IsoPs have been shown to increase in disease settings characterized by ischemia and reperfusion (I/R), including patients undergoing procedures as cardiopulmonary bypass grafting, thrombolysis, organ transplantation or embolectomy [86]. Increased levels of urinary or plasma F_2_-IsoPs have were reported in patients with chronic lower limb ischemia [87], following ischemic stroke [88,89], and after aneurysm rupture [90].

It is well understood that I/R elicits ROS formation. In 2020, Karlis et al. [91] evaluated F_2_-IsoPs reported the results from fourteen studies; only one study involved human subjects, while the remaining thirteen were experimental animal studies. The most important evidence is that plasma F_2_-IsoPs increase in the early post-resuscitation period and seem well-correlated with the burden of I/R injury [91]. In particular, F_2_-IsoPs levels increase as early as 5 min after experimental I/R injury in animals, with a peak at about 2 h and an attenuation at 4 h [86]. In a human model using suprasystolic inflation of an arm blood pressure cuff to safely induce localized forearm I/R, Davies and colleagues showed that plasma F_2_-IsoPs increase 15 min post-I/R and remain elevated for at least 3 h [92]. Moreover, the observed increase in F_2_-IsoPs is higher in healthy older (ages 62–81) than in healthy young adults (ages 20–33); in addition, F_2_-IsoPs levels remain elevated in the older population for a longer period of time [92]. Interestingly, in another study comparing physically fit older adults with unfit age-matched controls, classified based upon maximal oxygen uptake and maximal leg power, the F_2_-IsoPs response to forearm I/R was lower in the physically fit group [93]. This observation suggests that physical activity can have some beneficial effects on oxidative stress [93].

## 3. Cardiokines

A progressively increasing number of pathophysiological and clinical studies have been published in the last 5 years with the aim of identifying circulating biomarkers that are not only strictly related to oxidative stress mechanisms, but that are also able to monitor onset, progression, and treatment response of inflammageing in patients with cardiac disease [3,4,14,45,46,94,95,96,97,98,99,100,101].

The term “cardiokine” (or cardiomyokine) has been used by some authors to describe proteins or peptides secreted from any cell type in the healthy, stressed or diseased heart, that have autocrine/paracrine, and potentially endocrine functions [94,102,103]. Natriuretic peptides, C1q/TNF-related protein 9 (CTRP9), some interleukins (IL), and two Growth Differentiation Factors (GDF-15 and GDF-18) are probably the most studies cardiokines (Table 3) [94,102,103,104,105,106,107,108,109,110,111,112,113,114,115,116,117,118,119,120,121,122,123,124,125,126,127,128,129,130,131,132]. Several experimental and clinical studies have stressed the fundamental role of these cardiokines on cardiovascular diseases as well as in pathophysiological mechanisms of inflammageing [3,4,49,94,102,103,104,105,106,107,108,109,110,111,112,113,114,115,116,117,118,119,120,121,122,123,124,125,126,127,128,129,130,131,132]. Interestingly, some of cardiokines reported in Table 3 can modulate peripheral metabolism, such as natriuretic peptides (ANP, BNP and CNP), GDF-8 (also named myostatin), GDF-15, and CTRP9 [103].

From a pathophysiological and clinical point of view, it is important to note that all the cardiokines are released in blood by several tissues (not only by myocardial and vasal tissues), with the single exception of cardiac natriuretic peptides, ANP (Atrial Natriuretic Peptides) and BNP (B-type Natriuretic Peptides), that are peptides predominantly produced by cardiomyocytes [12,16,104]. For this reason, ANP and BNP are usually indicated as cardiac-specific biomarkers, rather than cardiokines [12,16,104,133,134].

### 3.1. GDF-8 (Myostatin)

Recent studies indicate that the cellular components of skeletal muscle are important sites for the release of proteins and peptides called “myokines”, suggesting that the skeletal muscle plays the role of a secretory organ [135]. Myokines may have many biological functions, including autocrine, paracrine and/or endocrine effects [135]. Accordingly, some myokines may affect complex multi-organ processes, including skeletal muscle trophism, metabolism, angiogenesis and immunological response to different physiological (physical activity, aging, etc.) or pathological states (cachexia, dysmetabolic conditions, chronic inflammation, etc.) [103,135].

The myokine myostatin (GDF-8) is a member of the transforming growth factor-beta (TGF-β) superfamily that is highly expressed in skeletal muscle [136], but also in myocardium and adipose tissue as basal expression [137]. The most important physiological function of myostatin is to regulate the mesenchymal stem cell proliferation and differentiation [136]. Indeed, in mice lacking the myostatin gene show decreased body fat and a generalized increase in bone density and strength [136]. The increase in bone density is observed in most anatomical regions, including the limbs, spine, and jaw, and myostatin inhibitors have been observed to significantly increase bone formation [136]. From a pathophysiological point of view, myostatin is expressed and secreted in skeletal muscle in response to stimulations, including oxidative stress or inflammation [137].

Considering the pathophysiological relationships between myostatin and cardiovascular diseases, the progression of heart failure is associated with increased expression of myostatin in the myocardium, skeletal muscles, and white adipose tissue, and elevated levels of this myokine were found in the peripheral blood [107,138]. In particular, increased levels of cardiac derived myostatin act in an endocrine fashion on skeletal muscle to reduce muscle mass, in this way inducing a progressive skeletal muscle atrophy in patients with chronic heart failure [105,106,107]. Chen et al. [139] investigated the association between serum myostatin and the severity and prognosis in 288 patients with chronic heart failure and 62 healthy controls. After 51-months follow-up, non-survivors (*n* = 173) had significantly higher serum myostatin than survivors (*p* < 0.01). Moreover, patients in the high tertile myostatin group had lower survival rate (73.95% vs. 93.75%; *p* < 0.05) and higher rehospitalization rate than those in the low tertile group, and Cox regression analysis showed that serum myostatin was an independent predictor of mortality [139].

A recent study reported that serum myostatin concentrations positively correlated with muscle mass and strength in 102 patients with Type 1 acute myocardial infarction [108]. Moreover, univariate analysis showed that patients with lower myostatin levels had higher mortality rates. Receiver operating characteristic curve analysis revealed that lower myostatin levels were associated with hospital mortality; while multiple logistic regression showed that higher serum myostatin levels were associated with reduced hospital mortality when adjusted by beta-blockers use (OR, 0.228; 95% CI, 0.054–0.974; *p* = 0.046) [108].

Although the relevant pathophysiological role of myostatin in patients with heart diseases (especially chronic heart failure) is clearly demonstrated [94,103,105,106,107,108,109,137,138,139,140], there are some doubts about the physiological role of this myokine in healthy subjects, in particular regarding the circulating levels related to age and sex in healthy individuals [140]. Morikaki et al. [141] reported no correlation between the circulating levels of myostatin, measured by a commercial ELISA method, with age and sex in 247 Japanese community-dwelling middle-aged and elderly adults (97 men, age: 75.0 ± 8.9 years;157 women, 73.9 ± 8.1 years). On the contrary, Barrios-Silva et al. [142] reported that age is negatively correlated (*p* = 0.02; R^2^ = 0.053) with total myostatin measured in heparinized plasma samples of 88 healthy individuals (range 18–68 years of age, *n* = 88, 39 males). It is important to note that the authors modified the commercial ELISA method used in the study in order to improve the assay specificity and to measure both total and free myostatin [142].

Indeed, myostatin in plasma can be found in two different states: free (unbound) or bound to some plasma proteins. Furthermore, the gene encodes a 375AA pre-pro-protein which is proteolytically processed by a protease, cleaving the NH_2_-terminal, or “pro-domain” portion of the molecule and resulting the active COOH-terminal dimer of myostatin (molecular mass about 25 kD). As in the bound status the biomarker shows a lower biological activity than in the free status, it is important to have a specific measure of free and total (bound + free) biomarker concentrations in order to have a better estimation of the biologically active myokine [142]. Furthermore, immunoassay methods may be affected by some peptides or proteins with structural, and potentially functional, similarity to myostatin, such as activin A and growth and differentiation factor 11 (GDF-11) [142,143].

Bergen et al. [144] developed a specific and sensitive new assay method based on liquid chromatography with tandem mass spectrometry (LC-MS/ MS), with the aim of more accurately measuring concentrations of free active myostatin in 80 younger (< 40 years), 80 older (>65 years), and 80 sarcopenic older women and men. The authors reported the following results: 1. older women had 34% higher circulating concentrations of myostatin than younger women; 2. per unit of lean mass, both older and sarcopenic older women had >23% higher myostatin levels than younger women; 3. younger men had higher myostatin concentrations than older men with and without sarcopenia; 4. younger men had approximately twofold higher concentrations of myostatin than younger women; 5. older women and sarcopenic older women had significantly higher relative myostatin levels than the corresponding groups of men; 6. circulating concentrations of myostatin exhibited positive, but not robust, correlations with relative muscle mass in both sexes. The authors concluded that myostatin may contribute to the higher prevalence of sarcopenia in women but acts as a homeostatic regulator of muscle mass in men [144]. 

The findings reported so far about the pathophysiological effects of myostatin suggest that more studies on factors affecting myostatin production and circulating levels are needed to better understand the relationship between this myokine and both aging and gender-specific differences. To achieve this goal, the development of sensitive and specific methods for routinary myostatin assay in the clinical laboratories is also needed.

### 3.2. GDF-15 (Macrophage-Inhibitory Cytokine 1)

Growth differentiation factor 15 (GDF-15) is a stress-responsive member of the Transforming Growth Factor β (TGF-β) superfamily, especially expressed and secreted in response to inflammation, oxidative stress and hypoxia [109,145]. GDF-15 is synthesized as a precursor protein that undergoes disulfide-linked dimerization. After a proteolytic cleavage at the N-terminal pro-peptide, the mature GDF-15 protein is secreted as a dimer with a molecular mass of about 25 kDa [109].

In health subjects, GDF-15 is weakly expressed in the major part of human tissues, but in some clinical conditions, characterized by inflammation, oxidative stress, and hypoxia the production of this cardiokine is increased in many cardiovascular cell types (also including cardiomyocytes), [109,145,146,147]. Indeed, several studies demonstrated that increased GDF-15 production is significantly associated with some metabolic (including obesity and diabetes mellitus) and cardiovascular diseases (including coronary artery disease and myocardial infarction), as recently reviewed in detail [109,110,111,145,146,147,148,149,150]. In particular, elevated circulating GDF-15 levels positively correlate with thickness of the posterior wall of the left ventricle, interventricular septum, and left ventricular mass [146,147]. Furthermore, several recent studies confirmed that circulating GDF-15 levels are significantly associated with increased cardiovascular risk (death and/or MACE) in patients with several cardiovascular diseases, including hypertension, atrial fibrillation, coronary artery disease, heart failure, myocardial infarction, and stroke [109,148,149,150,151,152,153,154,155,156,157,158,159,160,161,162,163,164,165,166,167].

Taking as a whole, this evidence strongly supports the hypothesis that GDF-15 is a reliable biomarker for cardiovascular risk in patients with metabolic and cardiovascular diseases [109,148,149,150,151,152,153,154,155,156,157,158,159,160,161,162,163,164,165,166,167]. The clinical relevance of GDF-15 as cardiac biomarker is also reinforced by the favorable analytical characteristics of the laboratory tests available for its assay. Indeed, as of recently, GDF-15 can be measured in patient blood with an electrochemiluminescence immunoassay (ECLIA) method using a fully automated platform, characterized by a high analytical performance [168,169].

### 3.3. CTRP (C1q/TNF-Related Protein) Family

Some recent studies have tried to explain and characterize C1q/TNF-related proteins (CTRPs) family as potential diagnostic and prognostic markers as well as therapeutic targets of obesity-related metabolic disorders (such as insulin resistance, type 2 diabetes) and cardiovascular disorders, as recently reviewed [94,103,112,113,170,171,172,173,174,175,176]. The CTRP family is a conserved group of proteins containing a structural similarity to adipokines, which are important regulators of appetite and satiety, energy expenditure, inflammation, blood pressure, hemostasis, and endothelial function [112,173,174,176].

The CTRPs are produced mainly in the epicardial adipose tissue, but heart, liver, kidney, and muscle tissues have been recognized as source of these proteins [174,176]. Several isoforms of CTRPs have been identified and each member of the 15 identified CTRP isoforms has a distinct function [112,173,174,176]. In particular, CTRP1, CTRP3, CTRP5, CTRP6, CTRP9, CTRP12, CTRP13, and CTRP15 have been reported to play a pathophysiological role in cardiometabolic diseases; more precisely, CTRP1 and CTRP5 promote a proinflammatory response, whereas the other family members have an opposite action in patients with cardiovascular diseases [174,175,176].

Among the other family members, CTRP9 has attracted the most attention following its discovery in 2009 [177]. The heart is the third richest organ for CTRP9 distribution, and cardiac function is significantly influenced by CTRP9; accordingly, CTRP9 should be considered not only an adipokine but also a cardiokine [94,102,103,174,178].

Considering the pathophysiological and clinical characteristics of CTRP9, two retrospective clinical studies reported conflicting results regarding the association between cardiac artery disease and CRP9 levels [179,180]. However, several clinical studies reported some protective effects of CTRP9 in patients with cardiovascular diseases, as recently reviewed [174]. In particular, Gao et al. [181] reported that circulating CTRP9 and CTRP3 levels are reduced in proportion to the severity of heart failure (according to New York Heart Association class) in patients with reduced ejection fraction. Moreover, multivariable regression analyses revealed that CTRP3 and CTRP9 levels were positively related with LVEF% (CTRP3, r = 0.556, *p* < 0.001; CTRP9, r = 0.526, *p* < 0.001) and negatively related with NT-proBNP levels (CTRP3, r = −0.454, *p* < 0.001; CTRP9, r = −0.483, *p* < 0.001) [181]. After follow-up for 36 months, the authors observed that CTRP3 or CTRP9 levels below the 25th percentile and adjusted for age, LVEF and NT-proBNP are predictors of total mortality (CTRP3, HR:1.93, 95%CI1.03~3.62, *p* = 0.042; CTRP9, HR:1.98,95%CI:1.02~3.85, *p* = 0.044) and hospitalizations (CTRP3, HR:2.34, 95%CI: 1.43~3.82, *p* = 0.001; CTRP9, HR: 2.67,95%CI: 1.58~4.50, *p* < 0.001) [181]. Another study reported that serum CTRP9 levels were significantly decreased in 128 patients with restenosis after cerebrovascular stent implantation [182]. Moreover, in this study CTRP9 levels were correlated with the change in nitric oxide, IL-6 and TNF-α levels, suggesting that this cardiokine may be a useful predictor biomarker for restenosis after cerebrovascular stent implantation [182].

However, to date, there are less and often conflicting results on the clinical usefulness of biomarkers related to CTRPs than other cardiokines or interleukines [94,103,112,174]. The lack of specific and sensitive assay methods, commercially available for all of the clinical laboratories, is almost in part the cause of this insufficient evidence on pathophysiological and clinical relevance of biomarkers related to CTRPs compared to other cardiokines (such as GDF-15) or interleukins (such as IL-6) or cardiac specific biomarkers (i.e., cardiac-natriuretic peptides and cardiac troponins).

### 3.4. Interleukine Family

Interleukins (ILs) are signaling proteins, included in the larger group of cytokines, regulating the inflammatory response by communicating proinflammatory and anti-inflammatory signals, such as cell proliferation, maturation, migration and adhesion [117,118,183]. ILs are predominantly expressed and secreted by leukocytes, but also by some other body cells [183,184,185]. The human genome encodes more than 50 interleukins and related proteins [183].

The fundamental role of the cytokines of the IL family in pathogenesis of several proinflammatory conditions was demonstrated many years ago; however, some recent studies have demonstrated that these ILs are also associated with relevant pathophysiological mechanisms that play an important role in cardiovascular diseases (Table 3) [114,115,116,117,118,119,184,185,186,187]. In particular, in the last 5 years, several systematic reviews including a meta-analysis have confirmed the significant role played by the ILs in the pathogenesis and prognosis of cardiovascular diseases [95,96,99,126,188,189,190,191,192,193,194,195,196,197,198,199,200,201,202,203]. The IL-1 superfamily (also including the sub-group IL-33/ST2L system) and IL-6 are the ILs most studied.

The IL-1 superfamily is a group of eleven cytokines (divided in three sub-family groups) sharing both pro- and anti-inflammatory activity [204]. IL-1α and IL-1β are the most studied members because they possess strongly proinflammatory effect mediated by the specific receptor IL-1R1. IL-1α and IL-1β have a natural antagonist IL-1Ra (IL-1Ra receptor antagonist), regulating IL-1α and IL-1β proinflammatory activity by competing with them for binding sites of their specific receptor IL-R1 [204].

Conversely, the IL-33 has an anti-inflammatory activity. For many years, the IL-1 receptor family member ST2 (proper nomenclature IL-1R4) was studied without knowledge of its specific ligand [204]. Only in 2005, a new interleukin of the IL-1 superfamily was identified as the specific ligand binding to IL-1R4, and then this new interleukin was named IL-33 [123,204,205]. IL-33 activates several immune cells, and it can also upregulate the release of cytokines such as IL-6 and IL-8 [126]. Major sources of ST2L and sST2 include endothelial cells of the aorta and coronary arteries, as well as some immune cells, such as T cells [126,204]. IL-33 has a unique specific receptor, named ST2. The ST2 receptor exists in two different forms: a transmembrane (ST2L) and soluble form, usually named Soluble Suppression of Tumorigenesis 2 (sST2) protein. The binding of IL-33 to its membrane receptor ST2 transduces the biological signal of the IL-33 to the cell nucleus, while the sST2 acts as a decoy receptor binding IL-33 in extra-cellular fluids and blood, to dampen its biological effects [126,204].

From an analytical point of view, all of the most clinically relevant ILs are usually measured with manual ELISA methods [99,103,114,115,116,117,118,119,206,207], while only the IL-6 can be measured with the electro-chemiluminescent immunoassay (ECLIA) Elecsys IL-6 method (Roche Diagnostics, Mannheim, Germany) [208,209] or the ADVIA Centaur Interluekin-6 test (Siemens Healthineers AG, Frankfurt, Germany) using fully automated platforms. The soluble protein receptor sST2 can be measured in plasma or serum samples using two high-performance immunoassay methods [210,211].

From a clinical point of view, in 2017 the results of two meta-analyses confirmed the role of ILs in the valuation of cardiovascular risk in patients with HF and the elderly general population. Aimo et al. [188] performed a meta-analysis, including seventeen studies with data on cardiovascular death from five studies, including 5051 patients. These authors reported that sST2 is a predictor of both all-cause and cardiovascular death in outpatients with chronic HF. The data of this meta-analysis support the use of sST2 for risk stratification in patients with stable chronic heart failure [188]. Li et al. [189] identified nine studies involving 9087 participants enrolled with the aim to investigate the association of circulating IL-6 levels with cardiovascular or all-cause mortality in the elderly general population (aged 60 years or more). The authors evaluated the association of circulating interleukin-6 levels with cardiovascular or all-cause mortality in the elderly general population (age ≥ 60 years). When comparing the highest with the lowest interleukin-6 levels, the pooled relative risk (RR) was significantly associated with all-cause mortality (1.49; 95% CI 1.33–1.67) and cardiovascular mortality (1.69; 95% CI 1.27–2.25), respectively [189]. Furthermore, subgroup analysis indicated the effects of interleukin-6 on all-cause mortality were consistently observed in sample sizes, region, durations of follow-up, interleukin-6 cutoff value and number of adjusted for covariates subgroups. The results of these two meta-analyses [188,189] strongly confirm that activation of proinflammatory ILs and inflammageing are both strictly related to pathophysiological mechanisms inducing senescence and HF, which the final common pathway of all cardiovascular disease [134,212,213]. Indeed, ageing is also the main risk factor for HF, and it is strongly correlated with age, with an incidence rate very low (<1%) in individuals under 50 years, but with a progressively increasing rate up to 30% in individuals with advanced age (>80 years) [212,213,214]. Interestingly, in a meta-analysis, Tabrizi et al. [194] evaluated the effects of statin treatment in 19,644 patients with metabolic disorders and increased cardiovascular risk according to the criteria of the National Cholesterol Education Program Adult Treatment Panel III on the circulating levels of some proinflammatory cytokines. The statistical analysis using the random effects model showed that statin use significantly decreases the standardized mean difference (SMD) values of IL-1 (SMD = −1.67; 95% CI, −1.98, −1.34; *p* < 0.001) and IL-16 concentrations (SMD = −8.35; 95% CI, −10.49, −6.22; *p* < 0.001) among patients with metabolic syndromes and related disorders. These data suggest that the measurement of some proinflammatory ILs (such as IL-1 and IL-6) may be also useful for monitoring the effects of pharmacological treatment in patients with metabolic disorders at higher cardiovascular risk.

### 3.5. Tumor Necrosis Factor (TNF) Superfamily

The tumor necrosis factor (TNF) superfamily is a group of transmembrane proteins containing the specific TNF homology domain, expressed predominantly by immune cells [215]. The superfamily contains 19 cytokine members that bind to 29 members of TNF receptor superfamily [215]. As a whole, the cytokines related to TNF superfamily have a relevant role in the regulation of diverse cell functions, not only immune response and inflammation, but also proliferation, differentiation, apoptosis and embryogenesis [215].

In particular, considering the pathophysiological role of TNF superfamily in the pathogenesis of cardiovascular diseases, the induction of necroptosis is the most relevant mechanism [216,217,218]. Necroptosis is a programmed form of necrosis or inflammatory cell death [218]. Conventionally, necrosis is associated with unprogrammed cell death resulting from cellular damage or infiltration by pathogens, in contrast to orderly, programmed cell death via apoptosis [218]. Even if necroptosis is thought to be especially involved in the first line of defense against intracellular infection, recent studies (including 4 meta-analyses) have indicated that this programmed form of necrosis can play an important role in a variety of cardiovascular diseases, such as atherosclerosis, ischemia-reperfusion injury, myocardial infarction, stroke and HF (Table 3) [99,100,103,115,127,128,194,218].

However, the experimental and clinical studies concerning the possible interrelationships among TNF cytokines (such as TNF-a), inflammageing and cardiovascular diseases are less numerous compared to other cytokines. A possible explanation may be that few specific and sensitive assays are suitable for the measurement of TNF-α in clinical laboratories, as recently reviewed in detail [219]. Furthermore, the assay methods for TNF-α assay in human are not yet harmonized and also may allow for false negative results in patients treated with anti-inflammatory drugs [219].

### 3.6. Transforming Growth Factor β (TGF- β) Superfamily

Transforming growth factor β (TGF- β) superfamily signaling pathways are ubiquitous and essential regulators of cellular processes including proliferation, differentiation, migration, and survival, as well as physiological processes, including embryonic development, angiogenesis, and wound healing [220]. The TGF-β superfamily of cytokines contains more than 30 structurally related polypeptide growth factors [220].

From a pathophysiological point of view, there are some studies especially concerning the TGF-β1 cytokine, a polypeptide member of TGF-β superfamily. TGF-β1 was first identified in human platelets as a protein with a molecular mass of 25 kDa [221]. It was later characterized as a large protein precursor (including 390 amino acids), which is proteolytically processed to produce a mature peptide of 112 amino acids [222]. TGF-β1 can play a role in many different pathophysiological processes related to the cardiovascular system, including cardiac development and angiogenesis, atherosclerosis, restenosis, hypertension, hypertrophic cardiomyopathy, myocardial infarction, and development of HF [103,129,130,131,132,220].

It is important to note that GDF-15 should in effect be considered as a member of the TGF-β superfamily. However, the GDF-15 cytokine is by far the most studied cytokine of this superfamily group, therefore the biological and pathophysiological characteristics of this cytokine were discussed in a specific previous paragraph in this review (Table 3) [109,110,111,145,146,147,148,149,150]. Indeed, the other members of this superfamily (also including TGF-β1) have been less studied probably because there are no specific and sensitive methods, able to accurately measure these cytokines in human biological fluids, that are available for clinical routine laboratories.

## 4. Cardiac-Specific Biomarkers

### 4.1. Cardiac Natriuretic Peptides

The natriuretic peptide system consists of three distinct endogenous peptides: Atrial Natriuretic Peptide (ANP), B-type Natriuretic Peptide (BNP) and C-type natriuretic peptide (CNP). All of the biological actions of the natriuretic hormones are mediated by three receptors: natriuretic peptide receptor (NPR)-A, NPR-B and NPR-C (a clearance receptor) [223,224]. It is generally believed that ANP is predominantly produced in the atria and BNP in the ventricles [104,223], while CNP is predominantly produced and secreted in the endothelial cells [224]. Accordingly, only ANP and BNP are considered to be cardiac-specific biomarkers [12,104,133,134].

NPs are synthesized as pro-hormones (proANP and proBNP), which are then split into two fragments at the time of secretion from cardiomyocytes: the longer fragment includes the inactive NH_2_-terminus peptides (i.e., NT-proANP and NT-proBNP), while the shorter one (i.e., COOH-terminus fragment) represents the biologically active hormones (i.e., ANP and BNP) [104,133,134,213,223]. Under physiological conditions myocardium tissues produces only a limited amount of BNP. Several mechanisms, such as ventricular hypertrophy, inflammation and fibrosis, stimulate BNP production and release from ventricular cardiomyocytes [104,223]. Indeed, the proinflammatory ILs and some adipokines (such as leptin, resistin, and visfatin) are able to activate the transcription factor NF-kB, and thus increase the production of BNP and related peptides from ventricular cardiomyocytes [224,225,226,227]. Plasma ANP is higher than plasma BNP in healthy adults [12,104,133,134,213,223]. Conversely, plasma BNP progressively increases more than plasma ANP as the myocardial function tends to decline in patients with cardiac disease [12,104,133,134,213,223].

There is a close pathophysiological link between increasing age, inflammation, activation of NP system, and cardiovascular disease, especially the onset and progression of HF [12,16,94,104,223,228,229,230,231,232,233,234,235,236]. Recently, McKechnie et al. [231] evaluated using multiple regression analysis the relationship of activation of natriuretic peptide system (evaluated by NT-proBNP assay) with baseline inflammation (evaluated by CRP and IL-6 assays) and incidence of HF in 3569 men followed for 16.3 years (aged 40–59 years at enrollment) and without prevalent myocardial infarction or HF. The results confirm that biomarkers of inflammation are significantly associated with an increased risk of incident HF in men [231]. NT-proBNP was associated with inflammation markers levels, more strongly with IL-6 than CRP. Moreover, the increased risk of HF associated with elevated IL-6 was only evident in men with high levels of NT-proBNP [231]. Therefore, activation of the NP system appears significantly linked to both inflammatory activity and risk of HF development in men >40 years [231].

NPs are commonly measured in the clinical laboratories using sensitive and specific immunometric assays using fully automated platforms [12,133,134,223,237,238,239], but some reliable Point-Of-care Testing (POCT) methods are also available [240,241]. In 2008, the quality specific recommendations for the analysis of BNP and NT-proBNP were reported by the committees of the National Academy of Clinical Biochemistry (NACB) and the International Federation of Clinical Chemistry and Laboratory Medicine (IFCC) [242]. Furthermore, in 2019, the educational document from the IFCC Committee on Clinical Applications of Cardiac Bio-Markers reviewed some important biochemical, analytical, and clinical aspects related to the measurement of NPs (in particular BNP and NT-proBNP) with a focus on HF [243].

For a routinary BNP assay, the use of tubes containing protease inhibitors (usually EDTA) is recommended [133,242]. For clinical trials and studies involving BNP, the appropriate storage conditions (including the use of some specific protease inhibitors and appropriate storage conditions) should be validated to mitigate any uncertainty regarding the effect of BNP degradation and the interpretation of results and outcomes [133,242,243,244]. Another important analytical issue is that several post-translational modifications (such as degradation and glycosylation) occur in both healthy subjects and patients with cardiac diseases producing a large heterogeneity among the active and inactive circulating NPs. These degraded or glycosylated products can affect the measurement of active peptide BNP by immunoassay methods [237]. 

Due to effect of possible interference with inactive peptides related to the active peptide hormone BNP, the IFCC C-CB guidelines recommend the use of mass units (ng/L, SI units) as opposed to pg/mL or pmol/L especially for BNP measurement in plasma samples [242,243]. Clinicians should be advised about the large differences among different BNP immunoassay methods, while NT-proBNP assays are more harmonized because use standards and materials made by the same manufacturer [237,245,246]. The IFCC C-CB guidelines encourage establishment of upper reference limits (URLs) stratified by age and sex for BNP and NT-proBNP assay methods [243]. Accordingly, all assays should report sex- and age-specific URLs by decade of life, using the upper 97.5th percentile to define normality [243].

### 4.2. Cardiac Troponin I and T 

Cardiac Troponin I (cTnI) and T (cTnT) are sarcomeric proteins, that are present with a specific aminoacidic protein structure in the myocardium cells (i.e., cardiomyocytes), which is different from the protein structures of the skeletal muscle troponins. The cTn are released in the setting of myocardial necrosis (i.e., myocardial injury) from injured cardiomyocytes [247,248]. Due to their specific aminoacidic sequences, cTnI and cTnT can be measured in blood samples of healthy subjects and patients with cardiac diseases using high-sensitivity immunometric (hs-cTnI and hs-cTnT) methods [249]. 

In 2018, the Fourth Universal Definition of Myocardial Infarction [250] established that a clinical condition defined “myocardial injury” is present when there is even a single hs-cTnI or hs-cTnT value above the 99th percentile URL. This document recommends that hs-cTnI and hs-cTnT should be measured in blood of all patients with suspicion of myocardial injury [250]. Furthermore, the term acute myocardial infarction (AMI) should be used when there is acute myocardial injury with clinical evidence of acute myocardial ischemia and with detection of a rise and/or fall of cTnI or cTnT values (preferentially measured with hs-cTn methods) with at least one value above the 99th percentile URL in the clinical setting of myocardial ischemia [250]. Accordingly, all of the most recent international guidelines recommend the use of hs-cTnI and hs-cTnT methods for the detection of myocardial injury as pre-requisite for the diagnosis of AMI in patients admitted to Emergency Department (ED) [251,252,253].

Circulating levels of hs-cTnI and hs-cTnT increase progressively after 55 years in asymptomatic men and women, enrolled in multicenter studies including large reference populations [15,16,249,254,255]. Many clinical studies and some meta-analyses have recently confirmed that some individuals apparently free from cardiac disease have hs-cTnI or hs-cTnT concentrations in the third tertile of the distribution values of biomarkers (i.e., still below the cut-off value of 99th percentile URL), who are at higher risk of earlier cardiac or non-cardiac mortality and/or rapid progression to heart failure [15,16]. Many aspects of mechanisms related to degradation, tissue release and elimination from the human circulation of cTnI and cTnT are still incompletely understood [247,248]. Myocardial injury may be caused by a variety of different mechanisms (including myocardial ischaemia, inflammatory and immunological processes, trauma, drugs and toxins) [247,248,250]. Moreover, myocardial necrosis is preceded by a substantial reversible (pre-lethal) phase [247,248].

Recent experimental and clinical evidence strongly supports the hypothesis that the plasma hs-cTnI and hs-cTnT concentration is a specific and stable index of the single healthy individual, strictly related to the physiological renewal of cardiomyocytes [247,248,249,256,257]. The mitosis rate of adult human cardiomyocytes in the healthy heart was evaluated to be only about 0.5% to 1% per year [258].

It is conceivable that cardiac troponins released into circulation because of physiological renewal of cardiomyocytes should be proportional to myocardial mass. This may explain why: a) the circulating levels of hs-cTnI and hs-cTnT are very low in adult healthy subjects (99th percentile distribution values ranging from 12 to 20 ng/L depending on the methods), corresponding to an estimated daily turnover of about 40 mg of myocardial mass; b) men have on average higher hs-cTnI and hs-cTnT values than women [247,248,249,255,256,257,258].

The close relationship between inflammageing and the progressive increase in hs-cTnI and hs-cTnt levels in older age should be emphasized. The senescence of myocardial tissue should be predominantly due the mechanisms related to the multi-component Senescence-Associated Secretory Phenotype (SASP), which is considered a fundamental hallmark of senescence, especially in tissue with very low cell turnover [4,259,260]. Senescent cells produce and release a variety of factors unified under the name of the SASP, including proinflammatory cytokines and chemokines, growth and angiogenic factors, matrix metalloproteinases, receptors/ligands, non-protein molecules (nitric oxide; PGE2; and ROS), and insoluble factors (collagens, fibronectin, and laminin) [259,261,262]. With aging, not only the production of collagen increases, but also the degradation becomes less effective [263], while the process of inflammageing produces a chronic cytotoxic effect on cardiomyocytes [4,6,259,261,262]. As a result, the most important effects of SASP mechanisms on myocardial tissue in older age are a progressive reduction in cardiomyocytes with a concomitant progression of myocardial fibrosis, which are the two fundamental hallmarks of heart senescence [259,260,261,262,263,264].

From a clinical perspective, the measurement of hs-cTnI and hs-cTnT should be considered an early, sensitive and specific biomarker of cytotoxic effects of inflammageing mechanisms on myocardial tissue, as well as the cognitive decline in older adults [15,16,265,266,267,268]. Considering both analytical and clinical characteristics commercial immunoassay methods available for clinical laboratories for an ideal biomarker (Table 2), hs-cTnI and hs-cTnT really represents the most ideal cardiac-specific biomarker compared not only to all cardiokines, but even to BNP/NT-proBNP [12,16,73,256,257]. However, the hs-cTnI commercial methods are not harmonized, and so these methods show different analytical performances, measured concentrations, and also cut-off values [249,254,255,269]. On the contrary, hs-cTnT is measured with only one commercial method with a cut-off value (i.e., the 99th URL value) corresponding to 13.9 ng/L [249,255,270].

### 4.3. Pathophysiological and Clinical Relevance of the Cardiac-Specific Biomarkers

Due to the higher cost of cardio-specific biomarkers compared with other laboratory tests, the clinical adequacy of the combined measurement of NPs and hs-cTn must be carefully evaluated [271,272,273,274]. However, considering the significant and independent information associated with cardio-specific biomarkers, the combined measurement may be convenient not only for the diagnosis, prognosis, and treatment in patients with heart disease, but also in patients at high cardiovascular risk of some extra-cardiac clinical conditions [16,237,271,272,273,274,275].

**Figure 1:**
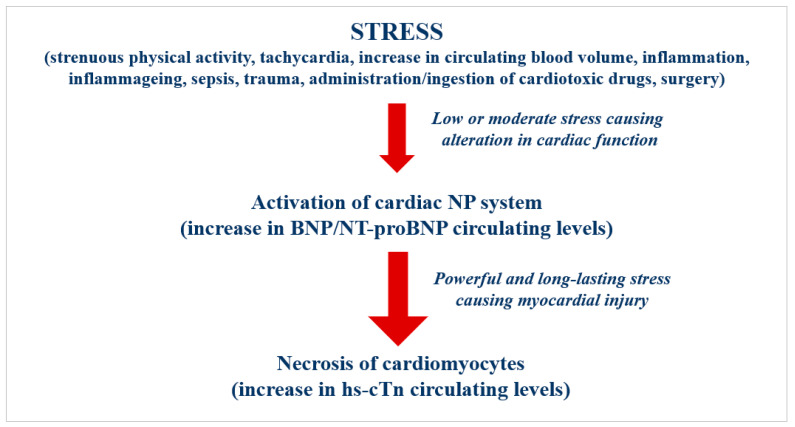
Relationship between stress and activation of cardiac peptide system and myocardial injury.

The rationale for the pathophysiological and clinical relevance of combined measurement of cardiac-specific biomarkers are highlighted in the Figure 1. The combined measurement of NPs and hs-cTn should allow to identify more easily these individuals who have a higher cardiovascular risk [16,270]. Indeed, NPs and hs-cTn have different but complementary characteristics [274]. Concentrations of NPs and hs-cTn are differently affected by the mechanisms causing cardiac dysfunction and/or cardiomyocytes damage [12,212,213,237,274]. Any increase in circulating levels of both cardiac-specific biomarkers demonstrates that the stress mechanisms have already caused relevant alterations on cardiac function (increase in BNP/NT-proBNP) and also a myocardial injury (increase in hs-cTnI and hs-cTnT) [250,274].

### 4.4. The Potential Role of Cardiovascular Risk Screening in the General Population Using Cardio-Specific Biomarkers

Recently, Francesco Salvatore suggested that paradigm of aging as the cause of frailty calculated as based only on the progression of chronologic age should be abandoned [276]. In particular, he suggested that some parameters should be identified and periodically monitored throughout an individual’s life to statistically detect or trend deviations that may harbinger a disease [276].

Indeed, laboratory medicine plays a key role in monitoring health status, because it is able to monitor aspects of preventive medicine in single individuals. The great experimental and clinical evidence accumulated in the recent years has clearly demonstrated that early detection and counteraction of inflammageing is a promising strategy not only to prevent cardiovascular disease, but also to slow down the progressive decline of health that occurs with ageing [2,3,4,5,6,7]. It is conceivable that beneficial effects of counteracting inflammageing should be most effective in the early stages, when the compensatory capacity of the organism is not completely exhausted. Early interventions and treatments require early diagnosis using reliable and cost-effective biomarkers. Several studies demonstrated NPs and hs-cTn should be considered early and sensitive cardiac-specific biomarker able to make evident the cytotoxic effects of inflammageing mechanisms on myocardial function and tissue [12,15,16,94,104,223,228,229,230,231,232,265,266,267,268].

Six meta-analyses, published from 2016 to 2022, have demonstrated that apparently healthy individuals of the general population with hs-cTnI and hs-cTnT values in the upper tertile of the biomarker distribution have a significantly worse cardiovascular outcome [14,277,278,279,280,281]. In particular, the results of the MORGAM/BiomarCaRE study supported the hypothesis that repeated measures of hs-cTnI or hs-cTnT are able to detect individuals in the general population at higher risk of MACE [282]. This study enrolled a Danish population (3975 participants, with an age at baseline of 30–60 years, 51% female, apparently healthy) followed with a total of 26 years (from 1982 to 2009). The hs-cTnI values were measured in samples collected in three series every 5 years [282]. Even if the median concentration of hs-cTnI in this population increased less than 1 ng/L (i.e, from 2.6 ng/L to 3.4 ng/L) over a 10-year period, however, this slight change in hs-cTnI was able to predict a significant increase in the 10-year cardiovascular risk (HR of 1.31, 95% CI 1.15–1.48) [282].

Considering this experimental evidence [14,277,278,279,280,281,282], two recent expert documents strongly suggest that hs-cTnI and hs-cTnT should be measured in the general population to detect early symptomatic individuals at higher risk of progressing to symptomatic HF or developing MACE over ≥6 months, such as patients aged >55 years and with comorbidities [15,16]. Accordingly, some clinical studies specifically evaluating the cost-benefit of a screening in the general population should be promoted in order to identify individuals at high-risk of progression toward symptomatic heart failure, by using the hs-TnI and hs-cTnT methods. Furthermore, the screening programs of cardiovascular risk stratification and prevention strategies incorporating hs-cTn requires further investigation to define the optimal target populations, timing of measurement, and preventive interventions.

## 5. Take-Home Messages

Experimental and clinical studies are essential to develop early-life biomarkers that efficiently identify individuals who are at high risk of developing accelerated heart and vascular damage, with the ultimate goal of improving primary prevention and reducing the health care and socioeconomic impact of age-related cardiovascular disease.

The measurement of cardio-specific biomarkers (NPs and hs-cTn) should allow for the easier identification of these individuals who have a higher cardiovascular risk compared to classical assay of oxidative stress and cardiokines [12,16,73].

Considering both analytical and pathophysiological characteristics, hs-cTnI and hs-cTnT really represent the most ideal cardiac-specific biomarkers compared not only to all cardiokines, but even to BNP/NT-proBNP [12,16,73,256,257].

The measurement of hs-cTnI and hs-cTnT should be considered an early and sensitive biomarker of cytotoxic effects of inflammageing mechanisms on myocardial tissue, as well as the cognitive decline in older adults [15,16,265,266,267,268]. 

Cardiovascular risk is apparently significantly higher in healthy subjects with concentrations of hs-cTnI and hs-cTnT in the upper tertile [15,16,277,278,279,280,281,282,283,284,285,286].

hs-cTnI and hs-cTnT can be measured in the general population to detect early symptomatic individuals at higher risk of progressing to symptomatic HF or developing MACE over ≥6 months, such as patients aged >55 years and with comorbidities [15,16].

There is still a lack of accurate studies that demonstrate the favorable cost/benefit profile of a cardiovascular risk screening with serial measurements of hs-cTn in the general population.

## Figures and Tables

**Table 1:** Pathophysiological mechanisms affecting cardiovascular longevity.

Oxidative stress
Inflammatory activation
Metabolic disorders: hyperglycemia, hyperinsulinemia, insulin resistance, dyslipidemia
Vascular disorders
Endothelial dysfunction, arterial hypertension, arterial stiffness
Genetic-epigenetic mechanisms
Telomere length DNA methylation

**Table 2:** Characteristics of an ideal biomarker (according to references [12,73]).

Acceptable to patient
Stability in vivo and in vitro
Adequate analytical sensitivity (functional sensitivity)
Good degree in reproducibility and accuracy
Easy to perform
Complete automation of assay
International standardization
Low cost
Low biological variation
Reference range and cut-off values tested for gender, age, and ethnicity dependence
Good diagnostic and prognostic accuracy
Cost-benefit ratio favorable

**Table 3:** Biological and pathophysiological characteristics of the most studied cardiokines.

Cardiokines	Related Conditions	References
Natriuretic Peptides (ANP, BNP, CNP and related peptides)	Cardiac stress, activation of neuro-immune-inflammatory systems, stretching of right atrium, hypoxia	[12,16,94,104]
GDF-8 (myostatin)	In heart failure, increased levels of cardiac derived GDF-8 act in an endocrine fashion on skeletal muscle to reduce muscle mass.	[94,103,105,106,107,108]
GDF-15 (macrophage-inhibitory cytokine 1)	Cardiac hypertrophy and chronic heart failure, ischemia/reperfusion injury, myocardial infarction	[94,103,109,110,111]
CTRP (C1q/TNF-related protein) family	Diabetes mellitus, coronary artery disease, ischemia/reperfusion injury, myocardial infarction, ischemic stroke	[94,103,112,113]
IL-1 family	Atherosclerosis, myocardial infarction	[103,114,115,116,117,118,119]
IL-6	Atherothrombosis, heart failure, atrial fibrillation	[99,103,115,117,118,120,121,122]
IL-33/ST2 pathway	Heart failure, inflammation, cardiac fibrosis	[99,103,123,124,125,126]
TNF-a	Coronary artery disease, Ischemia/reperfusion injury, heart failure	[99,103,115,127,128]
TGF-b1	Coronary artery disease, cardiac hypertrophy, myocardial infarction, atrial fibrillation	[103,129,130,131,132]

ANP: Atrial Natriuretic Peptide; BNP: B-type Natriuretic Peptide; CNP: C-type Natriuretic Peptide; GDF: Growth Differentiation Factor; CTRP: C1q/TNF-related protein 9; IL: interleukine; TNF: Tumor Necrosis Factor; TGF: Transforming Growth Factor.
